# Comparison of Clinical Outcomes after Platelet-Rich Plasma and Rotator Cuff Repair in High-Grade Intrasubstance Partial Rotator Cuff Tears

**DOI:** 10.3390/jcm12175554

**Published:** 2023-08-26

**Authors:** Grayson Poff, Edwin Spencer, Benson Scott, Robert Sleadd, John Broyles

**Affiliations:** Ortho Tennessee, Knoxville, TN 37923, USA; spencershoulder13@gmail.com (E.S.); benson.scott@orthotennessee.com (B.S.); robbsle2@gmail.com (R.S.); broylesjohn15@gmail.com (J.B.)

**Keywords:** clinical outcomes, PRP, intrasubstance rotator cuff tears, partial rotator cuff tears, platelet-rich plasma, rotator cuff repair, rotator cuff tears

## Abstract

Platelet-rich plasma injections have been shown to have many useful applications in various musculoskeletal pathologies. Research on the use of PRP for intrasubstance partial-thickness rotator cuff tears is lacking, although these tears have unique properties that may increase the efficacy of platelet-rich plasma injections. Patients with MRI-confirmed high-grade intrasubstance partial-thickness rotator cuff tears, that had failed traditional non-operative treatment, were offered either surgical repair (Group 1) or a single ultrasound-guided platelet-rich plasma injection into the tear site (Group 2). Patients were followed at 2 weeks, 6 weeks, 3 months, and a minimum of 2 years post-injection with ASES scores. A total of 25 patients received platelet-rich plasma injections, compared to 20 patients who had rotator cuff repair for intrasubstance tears in the last 3 years. The mean pre-injection ASES score for the platelet-rich plasma group was 53.2 and this improved to 92.9 at a minimum 2-year follow-up. The average convalescence period following platelet-rich plasma injection was 3.3 months. The average post-operative convalescence period for arthroscopic rotator cuff repair was 4.6 months. Both surgical repair and platelet-rich plasma injection into the tear site are equally effective in the treatment of high-grade intrasubstance partial-thickness rotator cuff tears, while platelet-rich plasma provides significantly shorter recovery time.

## 1. Introduction

Partial-thickness rotator cuff tears (PRCT’s) are a common cause of acute and chronic shoulder pain. Ellman classified partial-thickness rotator cuff tears by the depth of the tear. Grade 1 is less than 3 mm, Grade 2 is between 3 and 6 mm, and Grade 3 is greater than 6 mm in depth (high-grade), and these are further divided into articular (A), bursal (B), or (I) intrasubstance tears [1]. As described by Ellman, PRCT’s are a subtype of partial rotator cuff tears that occur between the bursal and articular layers of the rotator cuff [1]. This tear pattern poses a unique challenge to repair operatively, as many surgical techniques involve cutting into the bursal layer of the rotator cuff, creating a larger defect than was initially present. This research is specifically focused on high-grade intrasubstance PRCT’s. The management of partial-thickness tears is controversial and is evolving according to new clinical data [1,2,3].

The treatment of partial-thickness intrasubstance rotator cuff tears varies from non-surgical methods, including corticosteroid injections, NSAIDs, and physical therapy, to surgical repair [4]. When opting for arthroscopic rotator cuff repair, surgical repair with or without subacromial decompression (SAD) can be performed. Surgical repair usually involves taking down the intact bursal portion of the tear and repairing the cuff tissue to the greater tuberosity after the debridement of the interposed granulation tissue. Taking the bursal portion of the tear down creates a larger defect that can then be repaired as a bursal-sided PRCT.

A relatively new treatment option for partial rotator cuff tears is platelet-rich plasma (PRP). PRP has been proven to be a viable option for treating many musculoskeletal injuries [5,6,7]. Although given its anecdotal success, there is a paucity of data concerning the use of PRP in treating PRCTs. The use of PRP has been advocated for by clinicians of various specialties, and has shown some clinical success in various musculoskeletal pathologies [6,7]. Additionally, PRP can be subclassified by its leukocyte content—either leukocyte-rich (LR-PRP) or leukocyte-poor (LP-PRP) [6]. Data from Scarpone et al. support the use of LR-PRP to treat rotator cuff tendinopathy [6,7]. However, there are few data regarding the efficacy of PRP in the treatment of PRCTs, specifically intrasubstance tears.

We predict that an ultrasound-guided PRP injection into the intrasubstance PRCT will provide equivalent pain reduction and functional outcome in a shorter period of time when compared to surgical rotator cuff repair.

## 2. Materials and Methods

**Figure 1:**
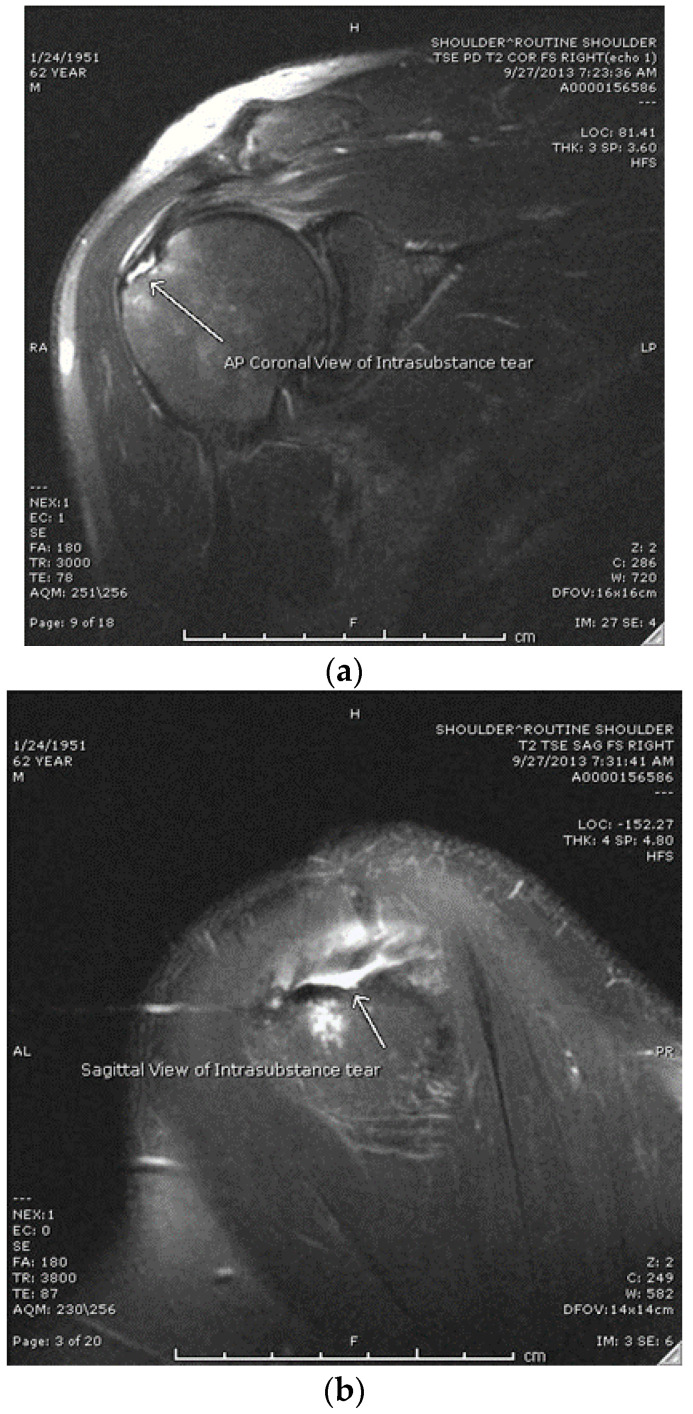
(**a**) Coronal T2 image from MRI showing an intrasubstance rotator cuff tear. (**b**) Sagittal T2 image from MRI showing an intrasubstance rotator cuff tear.

This study is a single-center retrospective cohort study/chart review analyzing functional and clinical outcomes from patients who were treated for an MRI-confirmed high-grade partial rotator cuff tear. Of the 233 charts reviewed, 188 patients were excluded due to having partial rotator cuff tear patters other than an intrasubstance tear (articular- or bursal-sided tears). Patients began receiving treatment in 2015 (when the first PRP injection was performed) through 2016, and data collection concluded at the 2 year mark for the final patients in 2018. The study was conducted in accordance with STROBE guidelines. American Shoulder and Elbow Society Index (ASES) scores and the length of convalescence were used as clinical measurements of success. Length of convalescence was defined as release to work/contact sports. Failure was defined as requiring surgery or an ASES score that did not improve by 15 points (MCID is 15 for ASES) [8,9]. The study population consisted of patients 15 to 80 years of age diagnosed with a high-grade intrasubstance partial rotator cuff tear of <2 cm (with MRI confirmation) who had failed non-operative treatment that included at least one of the following: NSAIDs, corticosteroid injections, or physical therapy, who then decided to undergo a platelet-rich plasma injection as an alternative to surgical intervention (Group 1). After confirmation of an intrasubstance tear via MRI (see Figure 1a,b), patients were presented with the option of a PRP injection vs. arthroscopic rotator cuff repair (RCR). 

**Figure 2:**
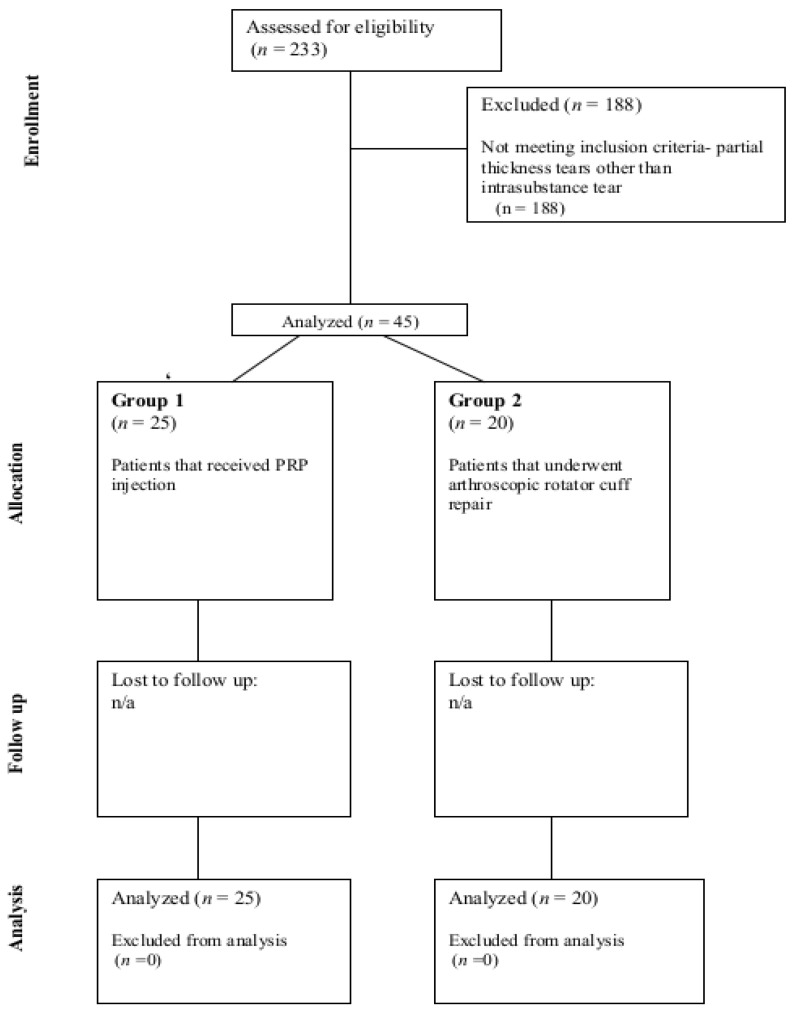
CONSORT flow diagram showing grouping, inclusion and exclusion.

Patients who elected to receive PRP injection were provided with the risks and benefits of PRP and RCR and signed an informed consent before proceeding with the ultrasound-guided PRP injection. Patients completed questionnaires regarding their pain and function at their 6-week, 3-month, 1-year follow-up, and 2-year follow-up. ASES scores were collected from each patient via a questionnaire. We also compared this to an age-matched cohort of operatively treated patients for the same type of tear who had elected to undergo arthroscopic RCR as opposed to PRP injection (Group 2). See Figure 2 for a CONSORT flow diagram of the patient grouping and inclusion. 

Data were then analyzed using a student’s *t*-test distribution to examine the difference in average percent improvement among the 2 groups. Testing the percent improvement, rather than simply the total increase in score, accounted for differences in initial ASES scores between the groups and ensured the *t*-test truly measured the improvement from the 2 treatment options rather than being skewed by the initial values. A student’s *t*-test was used to compare the ASES scores and the average time to release between the 2 groups.

Patients were contacted by phone or email if they did not show up for routinely scheduled follow-ups, and patients were contacted by phone or email to obtain 1- and 2-year post-treatment data. There was a 100% follow-up rate over 2 years, so no data were excluded or missing. Patients were only eligible if there was no other obvious pathology such as long head biceps, labrum or AC joint issues. In the surgical group, patients were excluded if any procedure other than RCR was performed. IRB Approval was obtained through Covenant Health in Knoxville, TN, USA.

### 2.1. Procedure

#### 2.1.1. PRP Injection

The PRP injection procedure was performed by a non-operative Sports Medicine physician. Each patient underwent a single PRP injection. Once consent was obtained for a blood draw, 30 cc of the patient’s blood was drawn in sterile fashion and spun down in an Emcyte centrifuge by Plymouth Medical in order to separate the blood products and concentrate the platelets, yielding 3–4 cc of leukocyte-poor PRP. Leukocyte-poor platelet-rich plasma (LP-PRP) was used instead of Leukocyte-Rich (LR-PRP), citing evidence of increased inflammation and post-injection pain associated with LR-PRP injection for tendon pathologies [9]. The same study also found no significant improvement in functional outcomes between LR- and LP-PRP. The procedure was performed using a 30 cc PRP kit from Plymouth Medical and prepared as per the manufacturer protocol. The lateral aspect of the shoulder is prepped and ultrasound is used to visualize the subacromial space and rotator cuff. Once the shoulder was anesthetized with Lidocaine, the injection was carried out under direct ultrasound guidance using a linear probe and PRP was injected into the intrasubstance tear identified on MRI imaging. Post-injection instructions were given to the patient, including avoiding anti-inflammatory medications and limited use of the shoulder for the next 3–5 days, then they began a PRP therapy protocol consisting of therapy twice a week for six weeks. The protocol is described in detail in Section 2.2.1. Patients were told to follow up in 3 weeks for a recheck.

#### 2.1.2. Arthroscopic Repair

All surgeries were performed in the beach chair position with general anesthesia and an interscalene block. A standard posterior portal was created and the glenohumeral joint was evaluated to ensure that there was no significant intra-articular pathology. The scope was then redirected into the subacromial space and anterolateral and posterolateral portals were created. The rotator cuff was then probed under direct visualization and the tear was localized by finding the “soft spot” at the cuff insertion that corresponded to the intrasubstance tear on the MRI. The tear was confirmed by inserting the probe through the bursal layer. Usually, serous-like fluid can be expressed through the perforation. Once confirmed, the bursal layer is sharply elevated off the greater tuberosity, exposing the intrasubstance tear. The interposed granulation tissue is removed and the greater tuberosity is lightly burred to expose the cancellous bone. The tendon was then brought down to the greater tuberosity using inverted mattress tapes and 1–2 suture anchors. The CA ligament was then released followed by acromioplasty if needed. 

### 2.2. Rehabilitation

#### 2.2.1. PRP Protocol

After the injection, the patient began a 6-week therapy protocol. In the acute phase (0–2 weeks), the primary goals include pain relief and minimizing swelling. NSAIDs were avoided during the acute phase. Patients typically remain at rest for the first few days, then begin a gentle range of motion exercises and passive joint mobilization coupled with modalities for pain relief. Strengthening is restricted to isometric exercises as tolerated. In the sub-acute phase (2–4 weeks), goals include isometric strengthening and stabilization for the rotator cuff. The light-activity phase (4–6 weeks), emphasizes isokinetic strengthening of the involved muscles. Stretching exercises progress to the active state and modalities remain consistent with the previous stage. Finally, the last stage (6 weeks onward) involves transitioning into more aggressive strengthening as tolerated. Patients are encouraged to participate in normal activities of daily life to tolerance. Functional restraining such as plyometrics and shoulder stability exercises are encouraged as pain allows. Return to sport is based on pain and function.

#### 2.2.2. Post-Operative Protocol

After surgery, patients are placed in a sling and encouraged to perform pendulum exercises as well as passive supine forward elevation and external rotation with the help of a family member or therapist. At the 2–4 week mark, they are cleared to begin periscapular strengthening as well as supine active assisted forward elevation. The next 6 weeks are aimed at increasing active ROM, beginning with supine active forward elevation and progressing to scaption, abduction, internal rotation, and external rotation. Patients also begin isometrics at this point. The last 6 weeks focus on deltoid and rotator cuff strengthening, resisted abduction, and neuromuscular control. Athletes may begin sport-specific activities at 3 months.

## 3. Results

The average age in Group 1 was 45, ranging from 16 to 62, with 40% female and 60% male. The average age in Group 2 was 56, ranging from 40 to 72, with 50% female and 50% male. Out of the 25 participants in Group 1, 24 reported successful outcomes for a 96% success rate. As seen in Table 1, the average primary outcome ASES score improved from 53.2 at baseline to 76 at six weeks, 85 at three months, 91.8 at one year, and 95.3 at 2 years post-injection. Table 2 defines the average dimensions of the rotator cuff tear for each group in the medial to lateral (M-L) and anterior to posterior (A-P). Group 1 had average tear dimesnions of 11.5mm A-P and 11.4mm M-L. Group 2 had average tear dimensions of 9.1mm A-P and 8.2mm M-L. As seen in Table 3, the average ASES score for Group 2 was 52.0 pre-op, 92.9 at one year, and 96.5 at 2 years post-op. As seen in Table 3, the average time from the injection to return to activity for Group 1 was 3.3 months, while the average time from surgery to return to activity in Group 2 was 4.6 months. The tear sizes were measured as the largest mediolateral defect on the coronal T2 MRI images and the largest anteroposterior defect on the T2 sagittal MRI images. The average mediolateral tear size for the PRP patients was 8.2 mm and the average anteroposterior tear size was 9.1 mm. The average mediolateral tear size for the surgically repaired patients was 11.5 mm and the average anteroposterior tear size was 11.4 mm. No significant difference was found between the ASES scores (*p* > 0.05), but Group 1 did have a significantly shorter convalescence period (*p* = 0.05).

Only one patient in Group 1 was considered a failure, defined as a symptomatic rotator cuff tear that required surgical intervention. A post-injection MRI revealed that the intrasubstance tear did not heal and this was confirmed at the time of surgery. A post-injection MRI revealed that the intrasubstance tear increased in size from 6.44 mm M-L and 6.7 mm A-P to 10.3 mm M-L and 8.7 mm A-P. The cuff also had more heterogeneous signal abnormalities throughout the tendon. This patient underwent an arthroscopic repair with two inverted mattress tapes taken down to a lateral row anchor with bursectomy and recovered successfully after 4 months. At the time of surgery the bursal portion of the cuff was so thin that the probe could be pushed through it. This bursal cuff was elevated and the typical granulation tissue in the defect that was derided. The exposed tuberosity was lightly decorticated and the cuff was repaired as described above.

In the surgically repaired patients, there were two complications. Two patients developed adhesive capsulitis that required a corticosteroid injection at 3 months after surgery. Both patients were non-diabetic females that recovered their full range of motion and function by 6 months after surgery.

Compared to surgical outcomes for the same type of tear, PRP injections showed a significant decrease in recovery time, with the time to return to pain-free, unrestricted activity being significantly shorter in Group 1.

## 4. Discussion

Among the patients who received PRP injections, mean ASES scores improved by 38.6 points, which is more than two times greater than the MCID [10,11]. Improvements in pain and functionality, as represented by the ASES score, suggest that the injection of LP-PRP into the intrasubstance tear promoted healing. These improvements are consistent with other studies regarding PRP injections for rotator cuff tendinopathy [10,12]. Arthroscopic RCR resulted in a 41-point improvement in ASES scores with no failures. There were, however, two complications of adhesive capsulitis that required corticosteroid injections that resulted in the resolution of symptoms and a full recovery by 6 months after surgery. This is similar to reports on surgical repair for PRCTs [13,14]. However, there was no statistical significance between the two groups (*p* = 0.646) when comparing ASES scores. In addition to clinical outcomes being similar between the two groups, the time to return to normal activity was significantly shorter for Group 1 by an average 1.3 months. This would suggest a clinical advantage to using PRP instead of arthroscopic RCR given the lack of significant difference between ASES score outcomes and a shorter convalescence period. The ASES score equivalence indicates that PRP is not inferior to RCR as a function of post-operative clinical outcomes at 2 years post-treatment.

The greatest benefit of arthroscopy over PRP is the ability to directly see if there is any other ancillary pathology. The authors have had two PRP failures since this study was concluded, both of whom had pathologies in the long head biceps tendon/pulley mechanism that were not appreciated on the MRI. The possibility of not appreciating other pathologies as a means of failure should be discussed with the patient.

To our knowledge, this is the first published report regarding PRP injections for intrasubstance PRCTs and it does suggest PRP as a viable treatment option for this particular type of tear. While there are limited risks associated with PRP injections, such as infection and post-injection pain and stiffness [9], these risks are typically less severe when compared to the risks of arthroscopic RCR. With regards to the data, we were able to confirm our hypothesis that PRP injections would provide equal improvement compared to RCR and provide a more rapid return to work or sport. It should be pointed out that this more rapid improvement could be, in part, because of the accelerated rehab protocol used in the PRP group.

Recent clinical studies regarding the use of PRP injections for various rotator cuff pathologies have shown promising clinical results [5,6,7]. To our knowledge, there are no published data evaluating PRP injections for intrasubstance PRCTs and evidence regarding surgical vs. non-surgical operative treatment for these types of tears has been inconclusive [7]. Research has failed to show that there is any difference between a subacromial anesthetic injection and a PRP injection in the treatment of PRCTs [9]. However, intrasubstance rotator cuff tears have the unique property of forming a sort of “pocket” within the rotator cuff tendon that could contain the PRP.

Platelet-rich plasma is an autologous blood product that contains numerous growth factors. These promote healing by stimulating biological pathways to facilitate the natural healing process. While Kesikburun showed that the injection of PRP into the subacromial space was not efficacious in the treatment of PRCTs, we are not aware of any studies that have specifically analyzed the efficacy of platelet-rich injections for high-grade intrasubstance partial rotator cuff tears by placing the PRP directly into the rotator cuff tendon [8]. Platelet-rich plasma falls under FDA’s Center for Biologics Evaluation and Research (CBER). PRP devices are approved by the FDA under 501 (k) clearance and Code of Federal Regulations Title 21 21CFR640.34 [5,10,11]. Despite the FDA’s approval, the injection is yet to be covered by insurance [12].

Interestingly, one patient in group 1 had a follow-up MRI at 1 year after the PRP injection related to a subsequent injury to the same shoulder. Comparison of the pre-injection and post-injection MRIs showed that the intrasubstance tear had healed and was comparable to the MRIs of patients within a 1-year post-operative window following arthroscopic RCR [15,16]. 

The primary limitation of this study is a lack of post-injection imaging to confirm the regeneration of rotator cuff tissue and healing of the intrasubstance tear. It is therefore possible that the PRP injections failed to heal the intrasubstance tear but other cellular mechanisms were able to relieve pain and render the tear asymptomatic. While the additional increase in functionality suggests that the rotator cuff tear did in fact heal, this cannot be confirmed. Additional limitations include a lack of ASES scores for the operative group until the 1 year mark. While the surgical cohort was included for comparison with the PRP treatment, the lack of ASES scores at the 6 week and 3 month marks provides limited use in comparing the rates at which the patients improved. The documentation of clinic visits included the date of return to unrestricted activity as well as VAS scores, which were similar to the data in Group 1. Post-operative data available for retrospective analysis were limited, but pain and range-of-motion measurements taken at post-operative visits suggest similar clinical improvement between the two treatment groups at both the 6 week and 3 month mark. Finally, the rehabilitation protocols utilized for each treatment group were unique to the specific treatment and surgical rehabilitation restrictions could have limited the potential for clinical improvement. In addition to normalized data collection across both treatment groups, randomized trials using the same treatment methods would yield stronger data, particularly utilizing controlled therapeutic protocols with coordinated rehabilitative milestones between the two treatments. Given the broad age ranges of both cohorts, the data can be generalized to a fairly wide patient population consistent with the age range observed within the study. However, only patients with IS PRCT’s were eligible, so the data cannot be generalized to other tear types or patients with ancillary pathology.

## 5. Conclusions

The injection of LP-PRP provided substantial clinical improvement for patients with intrasubstance rotator cuff tears comparable to that of arthroscopic rotator cuff repair. This suggests that PRP could serve as an alternative treatment option to arthroscopic repair with this particular pattern. Further investigation could aim to examine the efficacy of PRP injections in different tear sizes, as well as examining the effect of leukocyte concentration on clinical outcomes.

## Figures and Tables

**Table 1:** Demographics for each group.

	RCR	PRP
Age Range	40–72	15–62
Average Age	56	45
Male%:Female%	50%:50%	60%:40%

**Table 2:** Tear size dimensions for each group.

	RCR	PRP
A-P Measurement Average	11.5	9.1
M-L Measurement Average	11.4	8.2

**Table 3:** Mean ASES and Convalescence period lengths for both groups.

	RCR	PRP
Mean Pre-Op ASES	51.915	53.2
Mean Post-Treatment ASES	96.483	95.3
Mean Percent Improvement	44.568%	42.1%
Mean Convalescence Period Length	4.6 months	3.3 months

## Data Availability

The data presented in this study is available upon request to the corresponding author. Data was not made publicly available to maintain patient privacy.
